# ‘Why Bother?’ Skeptical Doubt and Moral Imagination in Care for People with Profound Intellectual Disabilities

**DOI:** 10.1007/s11013-025-09913-8

**Published:** 2025-05-09

**Authors:** Simon van der Weele

**Affiliations:** University of Humanistic Studies, Kromme Nieuwegracht 29, 3512 HD Utrecht, The Netherlands

**Keywords:** Care, Skepticism, Profound intellectual disability, Imagination, Empirical ethics

## Abstract

Caring well for people with profound intellectual disabilities is challenging. This challenge is often framed in terms of their complex needs and the ambiguity of interpreting these needs. Based on ethnographic fieldwork, this article argues that behind these challenges lies a more fundamental challenge of *doubt*: doubt stemming from uncertainties about the mind of the other, and thus about the purpose of care itself. Drawing on Stanley Cavell’s notion of skepticism, the article explores how this challenge arises and how caregivers grapple with it. The study finds that skeptical doubt always threatens care for people with profound intellectual disabilities, but often remains unseen. This is because caregivers deftly manage to ward off their skeptical doubt, by ‘placing people into life’: imagining the people in their care as participants in a shared human everyday life. The article tracks such exercises of ‘placing people into life’ to document how caregivers manage to retain faith in the purpose of their care. In this way, the article gives ethnographic texture to the challenge of caring well for people with profound intellectual disabilities and gathers clues for improving this care—which can also aid in improving care in other contexts of cognitive difference.

## Introduction

This article deals with the challenge of caring well for people with profound intellectual disabilities. Such people have ‘significant cognitive difficulties, with little or no apparent understanding of verbal language’ and ‘little or no ability to care for oneself’ (Mietola et al., 2017, p. 265). People with profound intellectual disabilities are thus wholly dependent on care and support from others in every aspect of life.

The challenge of caring well for these people is often framed in terms of their intensive and complex needs (Fitzgerald & Sweeney, 2013; Nieuwenhuijse et al., 2022; Tadema & Vlaskamp, 2010). Alternatively, the challenge is framed in terms of needs *interpretation*. Since people with profound intellectual disabilities do not express themselves verbally, interpreting their needs is rife with ambiguity (Grove et al., 1999; Nicholson et al., 2021; Phelvin, 2013; Porter et al., 2001; Vehmas & Mietola, 2021).

I argue that beneath these challenges lurks a more fundamental challenge of *doubt*: doubt stemming from deep uncertainty about the other’s mind and thus about the purpose and meaning of care itself. Shorn of a shared verbal language and faced with the alterity of persons with profound cognitive difference (Grøn, 2022; McKearney & Zoanni, 2018), it can be difficult to retain the belief that caring well *matters*; that it will make a difference how care is administered, as long as the bare minimum takes place. Hans Reinders (2008, p. 23) pointedly sums up this challenge with a rhetorical question: ‘why bother?’ Why bother caring well, when the people you care for do not consciously seem to receive it, nor ostensibly demonstrate much development through it?

This question is provocative and unsettling, but most scholars dismiss it as irrelevant to caring practice. Reinders himself considers the question of ‘why bother’ more philosophical than practical: only philosophers are detached enough from the everyday reality of caring for people with profound intellectual disabilities to question its moral value (see also Carlson, 2020; Curtis & Vehmas, 2021; Kittay, 2005, 2009, 2019; Vorhaus, 2018). The assumption seems to be that caregivers, firmly planted in practice, could not begin to doubt the meaning and value of their care.

The little available ethnographic research on care for people with profound intellectual disabilities does not fundamentally challenge this assumption. Patrick McKearney (2018) describes how caregivers at Christian charity L’Arche learn to regard people with intellectual disabilities as valuable and hence their care as meaningful. However, he does not probe whether caregivers can also *fail* to learn this—what happens when the challenge of doubt *does* creep in. Simo Vehmas and Reetta Mietola et al. (2017, p. 179) portray group homes in Finland where everything beyond the bare minimum of feeding and bathing was considered ‘optional.’ This standard of care seems to betray the challenge of doubt, but Vehmas and Mietola mostly place the blame for this ‘warehousing mentality’ on the care *system*; they are less attentive to how the challenge of doubt may arise for individual caregivers. Again, the assumption seems to be that doubt does not enter care.

I suggest that this assumption is mistaken; that the challenge of doubt constantly lurks in the background of everyday professional care for people with profound intellectual disabilities. This doubt occasionally surfaces in neglectful care practices or banter about the value of care. But usually, caregivers prevent this doubt from surfacing, as they are expert at resisting it. Hence, I argue we can observe the challenge of doubt most clearly in practices that thwart it—practices I shall call ‘placing people into life’.

To demonstrate this, I cast the challenge of doubt as an issue of skepticism: of uncertainty about other minds and our knowledge of them. I do so by reading my fieldwork alongside a philosophical and anthropological corpus on skepticism (Cavell, 1979; Das, 2007, 2020; Motta, 2023). Focusing on concrete, everyday care practices (Pols, 2015), I argue that caregivers ward off skepticism by exercising their moral imagination (Diamond, 1978, 1991). By imaginatively ‘placing people into life,’ caregivers fold the people in their care in a shared everyday life in which they do not easily partake.

My goals are to give ethnographic texture to the challenge of caring well for people with profound intellectual disabilities and to gather clues for improving this care. Indeed, I believe the practices of ‘placing people into life’ also shed light on caring well for other people with cognitive difference, such as people with dementia, whose humanity can similarly be subject to doubt (McKearney & Zoanni, 2018; Reinders, 2008; Taylor, 2008). Reading everyday care practices through the lens of skepticism spotlights the importance of moral imagination for care for differently minded people—a faculty that otherwise tends to go unnoticed.

## Methods

My analysis of care practices is informed by Jeannette Pols’s *empirical ethics of care* approach (Bredewold & Van der Weele, 2023; Pols, 2015, 2023; Pols et al., 2017; Van der Weele, 2024; Van der Weele et al., 2021). This approach ethnographically studies the implicit normativity of care practices: the ethical ‘background’ of ideals, values, preconceptions, and sensibilities informing care. It parses and infers this normativity by observing care practices and talking to interlocutors about them. The approach regards caregivers as actors who try to put something ‘good’ into practice (Mol et al., 2010). Analyzing these attempted ‘goods’ lets me articulate their ethical strivings toward ‘good’ care, as well as the ethical *challenges* to which their practices are a response (Van der Weele et al., 2021; Pols, 2019).

The particular ‘good’ I focus on appears in practices I below call ‘placing people into life.’ I tried to understand the moral logic of these practices by pinpointing the challenge to which they seemed a response. This analysis led me to the lexicon of skepticism. In what follows, I recount this journey in reverse, starting with the challenge of skepticism before detailing the work assistants performed to grapple with it.

My approach foregrounds care practices in their specificity. Hence, my analysis involves particular, sometimes seemingly negligible acts, gestures, and utterances I argue are nonetheless ethically charged. I present such practices to unearth new, ‘bottom-up’ ethical concepts for thinking (and doing) good care in everyday care practices, taking seriously the moral creativity of caregivers.

My empirical-ethical analysis draws on a multi-year ethnographic project, in which my colleague and I charted daily assistance for people with profound intellectual disabilities in the Netherlands (2019–2022) (Van der Weele & Bredewold 2021; Bredewold & Van der Weele, 2023, 2024). I shadowed residents with profound disabilities at group homes ran by six care organizations. I thus spent entire days with these residents and their assistants, alternatingly observing, interacting, and occasionally helping out assistants (McDonald, 2005). I also interviewed care assistants and relatives of the residents I shadowed to get a more holistic sense of what matters in their lives.

The residents I shadowed lived in a group home setting, meaning they had private quarters (e.g., room or apartment) in a building housing several housemates (typically up to eight) with whom they shared living and dining spaces. The residents’ days unfolded largely within these spaces. The rhythm of their days followed the rhythm of most people’s: breakfast, lunch, and dinner marked the course of the day, interspersed with activities like playtime, naptime, singing, and storytelling. They were aided by their assistants, usually two to four in each shift.

Since shadowing revolves around participation and interaction with one’s interlocutors, we could ask assistants questions about their activities. This was important, as much of group home life, in the absence of verbal communication, is instinctive and intuitive. I strove to deepen our conversations in subsequent interviews. Since I focus here on daily care practices, I draw exclusively from my observations and interviews with assistants. I use pseudonyms throughout.

## Two Helpings of *vla*: Scenes from Everyday Care

I begin by sketching two fieldwork scenes. The contrast between them spells out the challenge at stake in this article. Both involve a dollop of *vla*: a custard-like, dairy-based Dutch dessert.

The first scene occurred in a group home I call the Rotunda. Late in the morning, staff are preparing coffee for the residents. The mood is somehow mischievous: assistants are joking with each other and about the residents. ‘I’m not cleaning diapers today,’ one assistant declares. ‘They’ll just have to sit in their own shit for a day.’ Her joke is met with roaring laughter.

Another assistant, Pleun, finds some of yesterday’s apple pie in the fridge: a nice coffee treat, but there is not enough for each resident. After some humorous deliberation with her colleagues, Pleun decides to pair small pieces of pie with some chocolate-flavored vla, to increase the snack size. She mashes together the desserts in plastic bowls, one for each resident. She takes a bowl to resident Claudia and tentatively brings a spoonful to Claudia’s mouth. Claudia draws back her face. She squints her eyes and purses her lips. ‘What, don’t you like it?’, Pleun asks, in mock disappointment. She makes a sullen face; her colleagues laugh. (In fact, ten minutes later, Pleun offers Claudia the dessert again, who gobbles it up without complaint.)

In the world of Dutch desserts, apple pie and vla do not go together. You can pair two kinds of vla, maybe add some syrup (a *vlaflip*), but there is no custom of pairing vla with apple pie. Mashed together, they are hardly recognizable as dessert. Pleun is aware of this; her joke relies on this knowledge. So what does it mean that Pleun serves it anyway—and that her colleagues laugh at her doing so?

The second scene comes from a group home I call the Daffodil. I am in the living room with assistant Wander, who just took resident Eppo’s dessert from the fridge: a yellow tub of vanilla-flavored vla. The space is quiet; all assistants are concentrated on lunchtime. Wander opens the tub and holds it by Eppo’s face, to let him smell it. Wander explains that Eppo needs time to ‘get going’ before his meals; the smell helps him grasp it’s time to eat and activate his swallowing reflex. Wander hands Eppo his dessert by the spoonful. Swallowing is challenging for Eppo; each time he closes his mouth, part of his bite oozes out onto his right cheek. With each bite, Wander scoops up the spillage, and offers it to Eppo again. ‘You probably think this looks a bit gross,’ he tells me, almost apologetically. But he continues his task until the vla is finished.

There are striking differences between Pleun’s and Wander’s conduct and their work setting. Pleun is lighthearted and somewhat distracted; Wander is serious and engaged with Eppo’s experience. Pleun’s colleagues are jolly, even ridiculing; Wander’s are focused on caring. But most important to me is what they serve. While Pleun pairs vla with apple pie—an odd combination, if not plain wrong—Wander is serving vla *properly*: plain, by the spoonful, is simply how vla is eaten. Something brought Pleun to stray from this ordinary dessert custom. But what?

## The Threat of Skepticism of the Other

I want to argue that we can read Pleun’s conduct as inflected by a particular skepticism. So let me delineate this concept of ‘skepticism,’ which I take from Stanley Cavell. Skepticism, in the simplest sense, refers to doubt. I am interested in what Cavell’s commentators call ‘other mind skepticism’; that is, uncertainty about one’s knowledge of other minds—and even the reality of the other before me.^1^

For Cavell, doubt about other minds is a persistent feature of ordinary life: our lives are ‘lined’ with it, in Veena Das’s (2020, p. 58) metaphor. Skepticism takes root in the ordinary misunderstandings and uncertainties that mark our everyday lives with one another, in which even our most intimate knowledge about other people can be revealed to seem wrong or incomplete. Such moments can usher in a skepticism about our knowledge of the other: do I know you? Do I understand you? *Can* I understand you? (And also: can you understand *me*?)

At the core of such skepticism, Cavell located a distrust of what Wittgenstein (1953) called our ‘criteria’: our norms for the application of words by which we describe and come to know the world around us. Such distrust can emerge because our words are fragile. Words arise in and are bound up with specifically *human* lives and practices (what Wittgenstein called a ‘form of life’). For this reason, they can never secure certainty about reality outside of them (Laugier, 2015; Moi, 2002). We can thus come to doubt that the words we use make sense or express knowledge about others around us. As David MacArthur (2014, p. 3) puts it, ‘[w]hat is at issue is nothing less than our capacity to apply words to the world at all’.

Hence, at the heart of skepticism about other minds is doubt about the application of all these everyday words we use to describe the other’s mind, the other’s humanity, and indeed, our own: words like ‘person,’ ‘suffering,’ ‘joy,’ ‘hungry,’ ‘contented,’ ‘playful,’ ‘human,’ and so on. Such skepticism manifests itself in questions like: *Is he really in pain? Is she really suffering? Do you really care about me?* Or: *does she really enjoy dessert?* Such questions signal a desire for certainty about the life of the minds of others that our words cannot deliver.

Taken to their extreme, such skeptical questions unravel our ties to others. If skepticism has me question the other as *real* or *human*, it can also have me ignore any moral claim of another on me. Hence, skepticism is not just a feature of ordinary life, but also a threat to it. For this reason, Cavell considers mistaken doubts of the other a moral failure (Goodman, 1985). But he also insists that skepticism of the other cannot be dispelled with the certainty it craves. Certainty is precisely what our words cannot secure. Rather, responding to the other requires what he calls ‘acknowledgment’; a kind of ethical leap of faith to treat the other as real and human and like myself. This idea of acknowledgment harkens back to Wittgenstein’s (1953, p. 178) famous dictum: ‘My attitude towards him is an attitude towards a soul. I am not of the *opinion* that he has a soul.’ To acknowledge another means not to *know* for certain but rather to *act on* the other’s humanity.

Such acting on the other’s humanity involves engaging in the practices that humanity implies—practices like conversing, joking, hugging, greeting, and so on (Minar, 2024). It also involves using (‘projecting’) various words concerning human life. But importantly, there is nothing in our criteria that *warrants* treating the other as fellow human being. It is only by our *willingness* that we acknowledge the other as human and hence, as like me.

Acknowledgment is thus an act of moral imagination. It requires, as Cora Diamond puts it, an ‘imaginative understanding of what it is to have a human life’ (1991, p. 60). We need to imagine the other as sharing a particularly *human* life with us. In Cavell’s (1979, p. 421) words: ‘my identification of you as a human being is not merely an identification of you but with you.’ In everyday life, we enact such acknowledgment constantly, and hence constantly thwart skepticism’s threat to the fabric of the ordinary.

## Uncertainty and Profound Intellectual Disability

Back to the vla. Pleun served a dessert that would make most Dutch people wince. It is a dessert that is not *dessert*, neither delicious nor proper. I suggest this moment signals a brief appearance of skepticism; a moment when the lining of skepticism shows underneath the fabric of everyday life. Such skepticism resides in the sense that things do not really matter that much; the mash is somewhat funny, but good enough for Claudia. (‘Why bother?’) And importantly, such skepticism is not inevitable, as Wander’s commitment to getting vla ‘right’ shows.

Of course, Pleun is still caring for Claudia. What unfolds is not a failure of acknowledgment. In fact, by making a joke of Claudia’s response, Pleun is already folding Claudia back into a shared human world (more on this below). Nonetheless, Pleun’s willingness to give a ‘funny’ dessert—not to mention the bouts of laughter that preceded and followed it—hints at some uncertainty about the notion that Claudia truly participates in our shared everyday; that words like ‘dessert’ *apply* to Claudia. It is in this sense her mash contains the seed of skepticism. Skepticism does not fully enter the scene, but the door is set ajar.

The point I make here is delicate. The skepticism I claim is embedded in Pleun’s conduct is unspoken and in a sense also unspeakable. Indeed, part of the reason Pleun’s joke raised raucous laughter is probably because it contained an element of transgression. It is a joke assistants are not *supposed* to make—and most of the time, they do not. Hence, I emphasize my observation of skepticism is not a condemnation of Pleun or her care as such. Rather, I want to suggest that everyday care for people like Claudia is always ‘lined’ with the threat of this skepticism; Pleun’s dessert captures just one moment in which that threat becomes palpable.

Understanding why care for people like Claudia can be susceptible to skepticism requires a grasp of the alterity and uncertainty professionals experience in their caring practice. Eva Kittay (2019) writes that people with profound intellectual disabilities are often described ‘in the negative,’ through what they *cannot* do or be. And the qualities people with profound intellectual disabilities seemingly lack—rationality, language, autonomy—are also qualities many associate with the idea of humanity itself (Bogdan et al., 1989; Kittay, 2005; McKearney, 2018; Olsman et al., 2023; Taylor, 2008; Vehmas & Mietola, 2021). Cognitive difference as profound as this thus brings people to the margins of humanity and personhood; profound intellectual disability also means profound alterity (Van der Weele & Bredewold, 2024; Grøn, 2022; Kittay, 2005; McKearney & Zoanni, 2018).

Such alterity was palpable for my interlocutors, too, although not in these abstract terms. For assistants, the experience of difference was visceral, and constantly forced itself upon them, as an effect of what we might call the residents’ ‘opacity.’ Since residents could not express themselves verbally, the onus was on assistants to infer their moods, feelings, and experiences, without ways to ensure they were correct (McKearney, 2021; Olsman et al., 2021). To do this, assistants relied on nonverbal communication: on the residents’ cries, sighs, moans, gestures and movements, which assistants tried to read as ways in which residents sought to express themselves. Assistants did often manage to develop nonverbal ways of interacting that suited their residents and helped build relationships—especially through sound (the timbre and cadence of their voice) and touch (the sensation of their hands and fingers). Still, residents’ expressions were often ambiguous at best and assistants frequently disagreed about their meaning. Assistant Monica explains:

Knowing or asserting [facts] with Eppo, that can be very difficult. About food for instance. I might think, he likes chocolate porridge. And then a colleague can say: well, he never wants to eat chocolate porridge with me. And sure, it can be good for Eppo to have some variation [in porridge flavour] ... But we have to interpret a lot and also check with each other. What is a good life for him? What is really right for Eppo?

For Monica, residents’ opacity meant knowing things about them was difficult. Everyday interactions with residents were cycles of constant interpretation, beset by a persistent feeling of uncertainty (Bredewold & Van der Weele, 2024). All assistants I met felt this uncertainty. For instance, in another home called Sunrise House, I asked assistant Ferdinand to describe resident Dora. ‘That’s a profound question,’ he replied. ‘I’ve been working with her for a couple of years and still it’s a question for me whether I know her.’ When I asked him how he knows what Dora needs, he replied: ‘Truth be told, I *don’t* know.’

Anthropologists have analyzed such uncertainty in terms of ‘opacity,’ a concept denoting the difficulties involved in knowing the minds of others (Keane, 2008; Robbins, 2008; Robbins & Rumsey, 2008). This concept originates in the study of certain Pacific societies, in which an ‘opacity doctrine’—an assumption ‘that it is impossible or at least extremely difficult to know what other people think or feel’ (Robbins & Rumsey, 2008, p. 408)—is fundamental to the constitution of everyday ethical life. However, Patrick McKearney (2021) has argued that opacity claims also circulate in ethical life elsewhere; in his example, in intellectual disability care at l’Arche, a care organization he studied in the UK.

In my fieldwork, opacity similarly showed itself, albeit not as a claim, but as an anxiety. It manifested in the doubt assistants frequently expressed about whether their care was right and whether they really ‘knew’ their residents, like Monica and Ferdinand above. Such anxieties saturated caring in the group home, tinging everyday life with deep shades of uncertainty. It is clear how the threat of skepticism might arise in such circumstances, even if assistants also found nonverbal ways to engage in relationships with residents. Let me now give more ethnographic texture to how skepticism ‘lines’ ordinary life in the group home.

## The Threat of Skepticism in Everyday Care

Above, I observed an implicit skepticism in Pleun’s vla, because it displayed a certain carelessness. I observed such carelessness with some regularity. It showed in subtly inconsiderate moments, as when assistants discussed private information about one resident in the presence of other residents; when assistants left residents receiving tube feeding alone in the living room as the rest of the group ate together at the dining table; or when assistants startled residents by suddenly moving their wheelchair without warning. Very occasionally, assistants turned outwardly malicious, like when an assistant joked after hearing a resident’s distinctive yelling: ‘It’s like Animal Planet out here, 24/7.’ In such practices, residents no longer appear as people who can experience embarrassment, loneliness, or fright, or who are modest, excitable or indeed, human—as if these concepts do not *apply* to them (Vehmas & Mietola, 2021). ‘Why bother?’

Such carelessness was not the norm in these homes. But skepticism also surfaced in less dramatic fashion. One such way was the banter through which assistants occasionally mocked and even questioned their care. Take this scene from the Daffodil celebrating the feast of St. Martin, 11 November. The Dutch tradition is for children to go door to door with homemade lanterns, singing songs and asking for candy. The people of the Daffodil do the same, but now assistants are singing and holding the lanterns. The atmosphere is jolly, even cheeky. When given a bag of liquorish candies, assistant Isabel quips sarcastically, ‘Great, we’ll use that for liquorish porridge.’ (The joke is that Isabel has little use for liquorish, as most residents cannot properly chew it; but she also mocks the ubiquity of porridge in the home, a popular meal because it is fibrous and easy to eat.) And when handed a heavy tray of glass soda bottles, assistant Tanja points at two residents in wheelchairs saying, ‘Two strong men, they’ll have no problem carrying those.’ All assistants laugh heartily.

A second example comes from another celebration: the feast of St. Nicholas (*Sinterklaas*) at group home the Blackbird. Sinterklaas traditionally involves gift-giving, with children receiving presents from their parents. At the Blackbird, assistants give presents to the residents. As I watch assistant Paula wrap the hat she bought resident Adriaan, she jokes that the gift will go unappreciated, as Adriaan will not understand what the object is. ‘He’ll probably chuck it right away,’ she says, laughing.

I remember feeling surprised by these jokes. These assistants were riffing merrily on what usually remains unspoken: that residents seem to have no clue what they are doing and why they are doing it. The jokes betray some doubt about the meaning of their care—or at least awareness about the uncertainty of whether and how residents receive it. This is the face of skepticism.

One final way I observed skepticism in these homes was as an anticipated threat of *another’s* skepticism. Consider this scene from a fourth group home, Badger’s Burrow. Assistant Zora is readying tonight’s dinner, filling up the oven with trays containing ready-made meals. Asking if I can help, Zora instructs me to put Harry and Howard’s dinners on plates. As she instructs me, she points at two trays that need not be served on a plate. ‘Are those for the residents who get fed?’, I ask.

Immediately I notice a change in Zora’s mood. She turns away from the counter to look at me. ‘Animals you feed. *Humans* you give food,’ she says, her tone sharp. ‘It’s small, but I think this is very important.’ I ask Zora what she means. ‘I want to treat people the way I wish to be treated. So I don’t want to talk about feeding,’ she explains. ‘In care you can never do as much as you want. And it breaks your heart. But some things don’t cost money. Such as how you treat people. Treating people respectfully, and not like animals.’

Zora corrects my use of the word ‘feed.’ ‘Feed’ is what we do to animals; it is not appropriate to use in relation to humans. To speak of ‘giving food’ rather than ‘feeding’ is for Zora a matter of ‘treating people respectfully, and not like animals.’ Zora’s correction thus has moral implications: it suggests the words we use in the group home affect how we regard the home’s residents, and hence, how we treat them.

We might say Zora’s correction serves to affirm the humanity of the people she cares for: as an attempt to secure for herself and me the notion that before her was a person, by invoking words conceptually linked to humanity. Yet this affirmation also contains some disquiet. Zora seemed to anticipate some doubt about or challenge to the humanity of the residents on my part, which she wished to rebuke. Indeed, Zora later told me that she was mother to a young child with a mild intellectual disability, which had ‘changed her perspective on the world.’ Motherhood gave her a protective instinct against the bigotry suffered by people with intellectual disabilities. She explained this instinct had precipitated her correction. Here, skepticism enters the scene, not as carelessness, but as an anticipated threat.

Again, I acknowledge the delicacy of my analysis. These are not outright declarations of skepticism, but what I consider signs of its lurking presence. My point is that the conditions of uncertainty characterizing life in the group home render everyday care here as ‘lined’ with such skepticism—a lining of which I occasionally caught glimpses. In Zora’s correction, we already get some sense of how assistants attempted to keep this skepticism at bay. It is to this resistance against skepticism I now turn.

## Moral Imagination in Everyday Care: ‘Placing People into Life’

I return briefly to what Cavell considers antidotal to skeptical doubt: the act of acknowledgment, which for Diamond, requires moral imagination. Skepticism is not halted by acquiring more knowledge of others—this is the very desire that feeds skepticism—but by imagining the other as sharing a particularly *human* life with us: through an ‘imaginative understanding of what it is to have a human life’ (1991, p. 60). I take up this notion of moral imagination. If the care for people with profound intellectual disability is ‘lined with skepticism,’ how might moral imagination serve to thwart it? I probe this question with more scenes from my fieldwork.

The first occurred one day during lunchtime at the Daffodil. Assistant Annette is helping resident Barend to his porridge. She is about to stir in some cheese, but before she does, she holds it by Barend’s nose, to let him smell it. To my eyes, Barend barely notices the cheese; he keeps looking sideways, out the window, moving his head and arms in jerking movements. Annette is not bothered by Barend’s apparent disregard; she smiles as she lets him smell the food. ‘I suddenly notice how often I let the residents smell things,’ she tells me. She explains that she constantly thinks about what she calls ‘experience’ [*beleving*]: seizing moments to stimulate the senses of the people she cares for. ‘I want to place the residents in life in a way that suits them.’

I want to consider this expression, ‘place the residents in life.’ Later, in an interview with Annette’s colleague Monica, I asked her what she thought it means.

‘Placing in life’, I can imagine that… Such as when I was eating with that girl [a resident] today… You have to be [present] there [with her], but sometimes you also have to say: ‘take that cup’. And then it feels a bit like I place her in the moment or… well, ‘in life’ is quite big already, but in the moment.

Monica indicated that caring sometimes required more than being present for residents; sometimes you have to make the other present in the moment, too, for instance by having them ‘take that cup.’ She called this ‘bringing people along in the happenings of everyday life’—a phrase she had used throughout the interview and on several occasions when she talked to me during work. She meant she wanted to engage the residents she cared for in their own life: to make them part of the ordinary happenings they established together every day.

For Monica, bringing people along in everyday life comprised many things: allowing residents to experience certain sensations (such as smelling or tasting things); reading residents stories about the season they were in and the festivities taking place; involving residents in daily chores, such as doing the dishes; and any other practice that aimed at bringing the residents closer to a sense of shared everyday life. Whether residents were able to consciously *understand* their involvement had no priority. ‘If we wouldn’t do it, he [a resident] wouldn’t ask about it [*zou er niet naar talen*]… And still I am convinced that it does him good that we bring him along in the happenings of everyday life and of the year.’

These concepts circulating in the Daffodil—‘placing people in life,’ ‘bringing people along in life’—became helpful for me in pinpointing the ethical ‘good’ specific to the care practices I observed. It seemed to me many of these practices were in service of building and maintaining a sense of a shared everyday, in which assistants and residents lived their lives together. And the point was not to make residents *feel* part of their everyday life; it was rather to simply *make* them part of it. Establishing a shared sense of everyday life was an end in itself, regardless of the residents’ capacities to understand it or participate in it ‘fully.’

Importantly, this shared everyday also had to be a recognizably *human* one. Hence the assistants’ focus on meals, festivities, daily chores, songs, stories, seasons, shared jokes, and so on: all things that mark a human form of life. Recall Pleun’s joke at Claudia’s apparent disinterest in her dessert. She acted as though Claudia had rejected her offering and that this rejection hurt her. In this moment, she had Claudia participate in the joke, letting her appear as someone who was aware of the ‘wrongness’ of the vla-and-pie combination. In this way, the joke drew Claudia back into a shared human life—Claudia’s appetite for the dessert later that morning notwithstanding.

What was at stake, then, I believe, was imagining the residents as part of a shared human form of life. It is for this reason that I propose understanding ‘placing people in life’ as an act of acknowledgment through moral imagination: as Diamond puts it, it involves ‘an imaginative sense of shared humanity’ (1991, p. 55), by coming to see another as ‘someone who has a human life to lead, as do I, someone whose fate is a human fate, as is mine’ (1991, p. 59). It is in this gesture that assistants anticipated skepticism and warded it off, acknowledging the others in their care as real and human—and hence, their care as purposeful and meaningful. Above, I already described some such practices of this ‘placing in life,’ like involving residents in the ritual of traditional festivities, joking with them, having a meal with them, etc. In what remains, I flesh out two additional practices of ‘placing in life’ I have witnessed, which I call projection and narration.

## Projection

Consider again Zora’s correction of my use of the word ‘feed.’ Zora was teaching me about the application of this word in the group home. Another way of putting this is that Zora was correcting my *projection* of the word ‘feed.’ Cavell (1979, 2015) speaks of projection as the ways in which we use familiar words in new contexts to communicate with others. Projection is a function of the ‘fierce ambiguity’ of our words, the use of which Cavell calls both ‘tolerant’ and ‘intolerant’ (1979, p. 186). Tolerant, because words allow for creative projection into new and unforeseen contexts; and intolerant, because some projections will not be acceptable, will not communicate—our criteria do not allow for them. As Cavell puts it, ‘[a]n object or activity or event onto or into which a concept is projected, must *invite* or *allow* that projection’ (1979, p. 183). The projective affordances of a particular word cannot be predicted or known in advance. But our ability to *understand* one another’s projections (the projection’s communicability) requires that we share some sense of what the world is like and what is important in it; that we participate in the same form of life.

Now, Zora’s correction does not expose the *intolerance* of language to my projection of ‘feed’; after all, my projection *did* communicate something, but the message was somehow inappropriate. One could conclude that Zora’s correction was nonsensical. But the correction can also indicate that Zora was changing the *sense* of these words themselves for describing the goings-on in the group home. My projection was intelligible, but deemed inappropriate, because the animal connotations of ‘feed’ collided with Zora’s efforts to imagine and maintain the group home as belonging to a specifically *human* form of life. And by correcting me, Zora also initiated me in this form of life in which animals are ‘fed’ and humans are ‘given food.’ Here is one example of how projection serves to imagine the everyday life of the group home as human—in this case, by retooling the appropriate use of words important in group home life.

Here is another example, from Sunrise House. On an overcast day in late autumn, I join care assistant Jojanneke and four residents to work. Work consists in bringing yesterday’s garbage to a collection point outside the home. There is already some creative projection at work here: work [*werken*] typically implies carrying out one’s paid employment. Speaking of ‘work’ to describe this task is once again to imagine life in the group home as a human form of life, one in which people have jobs to which they go in the morning.

Huddled in our coats, we set off. Arjen and Claus walk independently, pushing the cart stuffed with bin bags; Eelco rides his electric tricycle; and Dora, the only resident in this home who is profoundly disabled, is in her wheelchair, aided alternately by Jojanneke and myself.

Arjen is reluctant to join work today. At several points during our walk, he lets go of the cart he is pushing and sits down on the pavement, arms crossed, looking sullen. Jojanneke attempts to alleviate Arjen’s recalcitrance by softly talking him into rejoining us, but Arjen remains unswayed. In a final gesture of protest, he throws his gloves on the pavement and then lies down flat on his back. Jojanneke swiftly picks up the gloves. Needing her hands to help Arjen get up, she places them on the tray attached to Dora’s wheelchair. ‘Here, you hold these, Dora,’ she says as she does so.

Consider Jojanneke’s use of the word ‘hold.’ Dora has a restless way of using her limbs. She repeatedly clenches her hands into various shapes and flings around her arms in unpredictable motions. Dora does not ‘hold’ objects in her hands in any typical sense—and certainly, she was not ‘holding’ the gloves Jojanneke placed on her tray. And yet Jojanneke’s projection made it so that Dora was holding the gloves and that Jojanneke had handed them to her. Her projection thus achieved two things: it made Dora a helping participant in the situation and also suggested a capacity for ‘holding’ I had not yet witnessed. In this way, Jojanneke’s imaginative projection invites us to acknowledge Dora as partaking in a shared human life.

One final example comes from the Daffodil and involves again the St. Martin’s feast. Recall that assistants had been joking about their resident’s obliviousness to the festivities. Yet even on this jolly occasion, the project of ‘placing people in life’ continued. Looking for a bag to carry the soda bottles, Tanja asked whose bags were still empty. ‘Leo can carry them,’ was the collective response—after which they asked *me* for the bag that *I* had been carrying as I was pushing Leo’s wheelchair. Here was another moment of creative projection, inviting us to imagine Leo as ‘carrying’ part of the evening’s bounty, and hence as a participant in this specific practice of our shared human life.

Note that many of these acts of projection are not directed at the residents themselves. In Zora’s case, the residents were not even present. These assistants used projections to fold the residents into the everyday, regardless of whether the residents *understood* it as such. The assistants did not strive to make residents feel included, but rather to secure to *themselves* the belief of their shared human life. This is why I read these projections as acts of acknowledgment: they do not seek epistemic certainty about the shared humanity of others, but assume it.

## Narration

Here are some scenes I witnessed on a typical morning in Sunrise House, in the hours after work and before lunchtime.

1. Ferdinand and the residents are preparing for the morning’s ‘coffee moment,’ waiting for one of Ferdinand’s colleagues to bring in the familiar cart with cans, cups, and a tin of biscuits. Suddenly, Dora lets out a few excited screams. Ferdinand directs his attention to her. ‘Yes, today is Saint John’s Day,’ he says. ‘But we won’t celebrate until tonight.’
2. After everyone has drank their coffee, Ferdinand reads the residents a story about Saint John’s Day. ‘On Saint John’s Day, we jump over a fire,’ he tells us. Eelco lifts up his index finger and mumbles incomprehensibly. ‘That’s right, there is only *one* fire,’ says Ferdinand, nodding toward Eelco.
3. Ferdinand gets up to haul away the coffee cart. He turns to Dora, who is mumbling wordlessly, intermittently bringing her left hand to her face and stroking her cheek. ‘You’ll keep an eye out, right Dora?’ He nods toward the other residents. ‘That they all don’t just walk off.’

Ferdinand engages the residents in the situation at hand through a particular mode of response to their gestures and their sounds. In an interview with me, he referred to this mode of response as ‘making a story out of it.’ He attempted to render the behavior of the residents intelligible by making it part of a narration of the situation as it unfolded. In an interview, he remarked:

Well, Dora often makes the same movement with her head, as if to nod ‘yes’… I also make use of that. So we’re sitting at the table, and I ask everyone, are you enjoying the food, does it taste good? And Dora nods ‘yes’ and then you have something fun and the other residents also respond to that positively. Because Dora doesn’t say anything, that’s true. But she really enjoys [the food]. And then I enjoy myself and I’m convinced that my enjoyment also reaches her… So even if she doesn’t say so, I interpret it and make sure she is doing well. I make her part of a story, part of what’s happening. If I don’t, I am just occupied with giving her food, tidying her up, but [I] try to do more.

Ferdinand characterizes his responses to Dora as acts of storytelling: assigning himself the role of an omniscient narrator, he interprets the various gestures and noises he observes and weaves them into a coherent story about the situation as it unfolds. The practice recalls what Cheryl Mattingly (2008) terms ‘narrative mind reading’: the practice of interpreting the motives that precipitate the actions of others, by drawing on shared cultural scripts and understandings. Such inferences, when ‘good enough,’ help everyday life run smoothly, as they allow us to respond adequately to others’ actions. Yet for Ferdinand, the point is not that these ‘mind reads’ of Dora’s behavior are *correct*. They are not about acquiring or securing more knowledge about Dora’s motives. The point is rather that his interpretations imaginatively allow Dora to be drawn into the situation and to regard her as actively shaping the situation. Hence, the question of Ferdinand’s knowledge of Dora—his doubt about the accuracy of his inferences—fades into the background.

Note also that Ferdinand professes he weaves these stories for *his* enjoyment, suggesting again that their purpose is not to uncover some truth about Dora’s inner life but rather to charge the situation (and Dora’s role) with significance for himself. Narration becomes a way of imagining Dora as an intelligible participant in the making of ordinary life in the group home. I think this is what Cavell would call acknowledgment.

Many assistants I met engaged in this project of storycraft, although not always self-consciously like Ferdinand. Once at the Daffodil, Tanja helped some residents to sausage sandwiches, remarking: ‘They’re true carnivores, these ladies. No vegetarians at the Daffodil.’ Here, she made a story out of the residents’ seeming taste for sausage, letting them become recognizable as non-vegetarians. And when Jantien, also at the Daffodil, read a story to the residents, she started every line with the name of one of the residents to whom she was reading: ‘Iris says that…’, ‘Helen tells us that…’. In doing so, Jantien created a story within a story, one in which not her, but the residents were relating a tale to one another and were engaged in conversation. In these ways, narration served to imagine residents as part of a shared everyday life.

## The Specificity of ‘Placing into Life’: Concluding Remarks

I have argued that the everyday care for people with profound intellectual disabilities is ‘lined with skepticism’ (Das, 2007, 2020). Assistants constantly grapple with their residents’ opacity, resulting in a mood of uncertainty that can foster doubt about the people in their care, and hence, about the meaning of care itself. By tracing this threat of skepticism, I wanted to show that the question of ‘why bother’ always lurks in the background of care for people with profound intellectual disabilities—even if it remains unnamed.

Why would most scholars dismiss such skeptical doubt as merely philosophical and hypothetical? The likely answer is that it simply feels *wrong* to accept that doubt may creep into care: that doing so amounts to a disrespect of the dignity of people with profound intellectual disabilities (Reinders, 2008; Vehmas & Mietola, 2021). To be sure, I share this concern. At times, examining skeptical doubt has felt dangerously close to making space for it.

Still, examining this threat of skepticism is valuable, as it lets us foreground the creative and imaginative everyday care practices that serve to *negate* the question of ‘why bother.’ These practices are geared at what, following my interlocutors, I have called ‘placing people into life.’ Such practices fold the people in their care into the everyday, by imagining them as participants of a shared human life. These practices only become noticeable if we dare to acknowledge the threat of skepticism to which they respond.

This notion of ‘placing people into life’ also helps develop an ethical vocabulary for ‘good care’ for people with profound intellectual disabilities. Practices of ‘placing people into life’ pursue the ‘good’ of acknowledging people with profound intellectual disabilities as people who matter, regardless of (in)capacities. Such acknowledgment seems essential to caring well for them, but is not given in caring practice (Bigby et al., 2012, 2015). Hence, the role of moral imagination in care needs recognition, appreciation, and discussion. The concepts I take from my interlocutors—‘placing people in life,’ ‘making a story out of it,’ etc.—provide a vocabulary to pinpoint the imaginative work of acknowledgment (see also Van der Weele, 2024). This vocabulary can also inspire other care contexts in which people face the possibility of having their humanity bracketed, like dementia care and disability care more generally (Driessen, 2018; McKearney & Zoanni, 2018; McKearney & Zogas, 2021; Taylor, 2008).

To flesh out the contribution that ‘placing into life’ offers, I now further specify it through comparison with two related anthropological themes: empathy (1) and personhood (2). In anthropological work on empathy (Hollan, 2008; Hollan & Throop, 2008; Throop, 2010), empathy concerns the attainment of ‘first-person-like knowledge of others’ through processes of intersubjective alignment (Hollan & Throop, 2008, p. 389). In this sense, empathy and ‘placing into life’ are both responsive to the difficulty of knowing others. However, the imaginative practices I tracked also differ from the dynamics of empathetic alignment explored by anthropologists. Even if empathy does not require ‘knowing everything that others know’ (Throop, 2023, p. 28), it still designates a responsivity to, and hence grasp of another’s mental state. Yet the practices I described precisely avoid the need for such a grasp. Establishing a shared a human everyday is an end in itself, and can be achieved without certainty about residents’ capacity to experience it or participate in it. In fact, insofar as some of these practices of ‘placing into life’ are wholly verbal, they intrinsically preclude residents’ understanding of them, and occur even in their absence. Hence, unlike empathy, ‘placing people into life’ addresses the problem of opacity not by interacting with and thus acquiring knowledge about the other, but simply by *assuming* the other’s fellow humanity.

Anthropologists of care have long thematized the relationship of care to personhood: what Elana Buch (2015, p. 281) calls the ‘formative potential of care to generate and sustain social persons’ (see also Buch, 2013; Driessen, 2018; Kleinman, 2009; McKearney, 2018; Mol, 2008; Taylor, 2017). This theme fits with ‘placing into life,’ which partially seems directed at conferring personality onto people with profound intellectual disabilities, to help them gain an identity with traits and preferences—what Hilde Lindemann (2002, 2014) calls ‘holding the other in personhood.’ Disability scholars have observed similar practices of identity-bestowal in care for people with intellectual disabilities (Bogdan et al., 1989; Reinders, 2008; Vehmas & Mietola, 2021). Yet distinctive about ‘placing people into life’ is its focus on the formation of a shared social world. Assistants let people participate in a recognizably human life, as to ‘enfold cognitively disabled people into the social’ (Friedner, 2018, p. 109). These practices do not just sustain persons, but also sustain a shared everyday *amongst* other persons. Hence, ‘placing into life’ adds to this theme of person-sustaining care the capacity of care to build and sustain a common social world; a variation of the worldbuilding aspect of care described by Maria Louw (2022).

This creation of a shared everyday is a notable achievement, but also a responsibility for caregivers and organizations. Vehmas and Mietola (2021, p. 3) observed a poor standard of care in many group homes they visited, for which they blamed a ‘mechanical, unimaginative care culture.’ The imaginative work I have described can invigorate such cultures. For this to happen, we need words to discuss such work. The vocabulary of ‘placing people to life’ can facilitate such conversation.

## Footnotes

1 Cavell’s distinction between ‘external world skepticism’ and ‘other mind skepticism’ is beyond this article’s scope. See MacArthur (2014) and Marco Motta (2023).
